# Effect of low-intensity pulsed ultrasound on scaffold-free ectopic bone formation in skeletal muscle

**DOI:** 10.3109/03009730903226659

**Published:** 2009-12-08

**Authors:** Munenori Watanuki, Koshi N. Kishimoto, Satoshi Kotajima, Sadahiro Iwabuchi, Shoichi Kokubun

**Affiliations:** ^1^Department of Orthopaedic Surgery, Tohoku University School of Medicine, SendaiJapan; ^2^Bio-medical Engineering Laboratories, Teijin Pharma Ltd., TokyoJapan

**Keywords:** Ectopic bone formation model, in vivo electroporation, LIPUS

## Abstract

**Background:**

Low-intensity pulsed ultrasound (LIPUS) is reported to have the effects of rapid appearance and early maturation of ossification in animal models.

**Method:**

We examined the influence of LIPUS on bone formation in C57BL/6J mouse muscle induced by gene transfer of BMP-4 expression plasmid. Electroporation was employed to transfer plasmid DNA. First, an *in vitro* study was carried out to confirm that LIPUS has no effect on the forced expression of BMP-4 gene transferred by electroporation into C2C12 cells. Next, the BMP-4 plasmids were injected into mouse calf muscles, and transcutaneous electroporation was applied. LIPUS (30 mW/cm^2^) exposure was performed daily for 20 minutes on one side of hind limbs (LIPUS side). The contralateral limbs were not exposed to LIPUS (control side). Muscle samples were collected at 7, 10, 14, and 21 days after electroporation. Soft X-ray films of muscles were taken, and areas of bone formation were measured. After pepsin solubilization of the muscles, calcium and total collagen content were measured.

**Results:**

Radiographical measurements showed significantly more bone formation in the LIPUS side at Day 10. The area of bone was the maximum in both sides at Day 14. The LIPUS side exhibited significant increase in the calcium content at Day 10. The total collagen content with LIPUS exposure was increased significantly over control at Day 10 and 21.

**Conclusions:**

According to these results, accelerated maturation of ectopic bone formation by LIPUS was confirmed at Day 10. Moreover, our results showed that LIPUS increases the total collagen content during osteogenesis.

## Introduction

Low-intensity pulsed ultrasound (LIPUS) is known to accelerate fracture healing (1) and soft tissue regeneration following injury, such as bone-tendon junction (2) and nerves (3). LIPUS has been found to stimulate the healing of fresh fracture (1), non-union, and delayed union (4,5) in clinical treatment. Cell culture studies showed that LIPUS could stimulate expression of Runx2, collagen, alkaline phosphatase, osteocalcin, insulin-like growth factor (IGF)-1, and transforming growth factor (TGF)-β1 in osteoblastic cell lines (6), fibroblasts (7), osteoblasts (7,8), and bone marrow stromal cells (9). A large number of animal studies have demonstrated the potential of LIPUS to enhance fracture healing, resulting in a much stronger and stiffer callus (10,11). In animal osteotomy and distraction osteogenesis models, LIPUS leads to the increase of bone mineral content, bone mineral density, peak torque, and stiffness as well as to the rapid appearance and maturation of the overall endochondral ossification processes (12,13). These animal osteotomy or distraction osteogenesis models, however, have a limitation. It is impossible to measure the correct size of fracture callus isolated from the pre-existing bone.

We have studied the bone induction using *in vivo* electroporation with plasmid expression vectors. Electroporatic gene transfer of bone morphogenetic protein (BMP)-4 expressing plasmid into skeletal muscle can induce bone formation without any carriers (14,15). BMPs can transform myoblasts and pluripotent mesenchymal cells into osteogenic cells *in vitro* (16) and *in vivo* (17). For *in vivo* bone induction, scaffold is thought to be necessary for the continuous delivery of BMPs and for cell migration (18). Gene transfer techniques make it possible to release gene products continuously and have been used as new drug delivery systems. Transfer of recombinant human BMP-2 (rhBMP-2) using an adenoviral vector to skeletal muscle cells, including myoblasts and myocytes, induced ectopic bone formation without any carriers (19,20). However, virus vectors have many problems such as immunologic reactions and an uncontrollable spatial range of infection. Electroporation could be a possible non-viral gene transfer technique to resolve these problems. Bone formation by electroporatic BMP gene transfer could be not only a possible clinical application but also an interesting experimental model for *in vivo* bone formation.

The objective of this study was to examine the influence of LIPUS on *in vivo* ectopic bone formation induced by electroporatic transfer of BMP-4 gene. We hypothesized that LIPUS may accelerate the maturation of ectopically formed bone and increase its amount.

## Methods

### Plasmid

A 1.6-kb mouse BMP-4 cDNA (a gift from Dr Hogan (21)) was inserted into the multiple cloning site of the pCAGGS expression vector (a gift from Dr Miyazaki (22)) (pCAGGS-BMP4). Plasmids were grown in *Escherichia coli*, extracted using HiSpeed Plasmid Maxi Kit (Qiagen, Hilden, Germany). Purified DNA was dissolved in Dulbecco's phosphate-buffered saline (Invitrogen, Carlsbad, CA) at 3.0 μg/μL. Its quantity and quality were assessed by optical densitometry.

### In vitro studies

C2C12 cells obtained from RIKEN (Tsukuba, Japan) were maintained in Dulbecco's modified eagles medium (DMEM) supplemented with 10% fetal bovine serum (Invitrogen, CA), 50 U/mL penicillin, and 50 mg/mL streptomycin. Trypsinized cells were dissolved in electroporation buffer (75% cytosalts; 120 mM KCl, 0.15 mM CaCl_2_, 10 mM K_2_HPO_4_ pH 7.6, 5 mM MgCl_2_; 25% Opti-MEM1) at a concentration of 2.5 × 10^6^ cells/mL (23). Plasmid solution was added into 600 μL of cell suspension. Electric pulses were applied through a 4 mm gap cuvette. Pulse settings were 450 V, 2 microseconds, and 2 pulses at the interval of 1 second. After electroporation, cells were recovered in the medium overnight.

#### Ultrasound stimulation.

The LIPUS exposure system (Teijin Pharma, Tokyo, Japan) was used to create LIPUS with temporal average intensities of 30 mW/cm^2^. The frequency was 1.5 MHz with a 200-microsecond tone burst repeated at 1.0 kHz. The pattern of the LIPUS signal employed in this study was essentially the same as that used in clinical practice (4) and in animal model experiments (24). The LIPUS exposure device was specially designed for use in a 6-well tissue cell culture plate (25). The transducers were set on a frame and immersed in a water-bath with a controlled temperature of 37°C, and the culture plate was located on the transducers. LIPUS was applied at 24 hours after gene transfer. Control samples were subjected to the same manipulations under the same conditions but without ultrasound exposure.

#### Quantitative reverse transcription polymerase chain reaction.

For quantitative reverse transcription polymerase chain reaction (RT-PCR), the cells were harvested at 1, 3, and 6 hours from the end of the ultrasound stimulation. Total RNA was isolated from each well using RNeasy Mini kit (QIAGEN Inc., CA). Single-stranded cDNA was synthesized using High-Capacity cDNA Archive Kit (Applied Biosystems, CA). Real-time quantitative analysis was carried out with ABI PRISM 7700 (Applied Biosystems, CA) and CYBR Green PCR Master Mix (Applied Biosystems, CA). Expression of BMP-4 was analyzed. Primer pairs for mouse BMP-4 are 5′-GATTCCTGGTAACCGAATGC-3′ and 5′-AAGTGTCGCCTCGAAGTCC-3′. Ct values of gene of interest (Ct(GOI)) were standardized by a Ct value of GAPDH (Ct(GAPDH)). Results were shown as -ΔCt= -(Ct(GOI)–Ct(GAPDH)).

### In vivo studies

This *in vivo* study was carried out with permission from the committee of animal experimentation. C57BL/6J male mice were purchased from Clea Japan, Inc. (Tokyo, Japan). Mice were maintained under specific pathogen-free conditions at the Institute for Animal Experimentation.

#### In vivo electroporation.

In vivo electroporation was performed on 9-week-old mice. The electrodes consisted of a pair of 99.95% pure tungsten rods (Nilaco, Tokyo, Japan). The diameter of the needle-shaped electrodes was 0.3 mm, and the distance between them was fixed at 5 mm. The mice were anesthetized by intraperitoneal injection of pentobarbital sodium (50 mg/kg). The pCAGGS-BMP4 solution (150 μL) was injected into the mid-part of the gastrocnemius muscle using a disposable insulin syringe with a 27-G needle. The electrodes were inserted into the gastrocnemius percutaneously immediately after injection of the DNA, and electroporation was carried out according to our previous report (14). Rectangular pulses of 100 V and 50 ms, at 1 pulse/s, were charged by a pulse generator (Electro Square Porator T820, BTX, Holliston MA) and monitored using a graphic pulse analyzer (Optimizor, BTX). Without interruption, three electric pulses were applied, followed by three more pulses of the opposite polarity. The bilateral limbs underwent the same procedures.

#### Ultrasound stimulation.

The parameter of LIPUS employed in this *in vivo* study was the same as in the *in vitro* study. Mice were anesthetized and placed on the holder. LIPUS exposure was performed daily for 20 minutes through a transducer on the lateral aspect of the gastrocnemius on one limb of each animal (LIPUS side). The mock transducers were placed on the contralateral limbs without LIPUS exposure (control side). The side of experiment was randomly allocated. Nine animals were killed by cervical dislocation at 7, 10, 14, and 21 days after electroporation (6 for quantitative tests and 3 for histological evaluation).

#### Radiographic analysis.

The gastrocnemius muscles encapsulated in fasciae were dissected out, and anterior-posterior X-ray film of gastrocnemius was taken at 25.0 kV and 3.0 mA, for 10 s (SRO-iM50, Sofron, Tokyo), using X-Omat TL film (Kodak). The X-ray films were digitized using an Epson ES2000 scanner (Epson, Tokyo, Japan), and areas of bone formation were measured using ImageJ analysis software 1.33 (NIH, Bethesda, MD, USA; http://rsb.info.nih.gov/ij/

#### Quantitative analysis.

The gastrocnemius muscles were dissected to trim off the attached adipose tissue and tendon, diced into cubes of 1 mm, and then dried in a lyophilizer for 6 hours. After the dry weight of the samples was measured by an electronic balance (TC-104, Denver Instrument Company, Arvada, CO), the samples were homogenized and then digested for 48 hours at 4°C with 1 mg/mL of pepsin (WAKO, Tokyo, Japan) in 7.5 mL of 0.5 M acetic acid containing 1.0 M NaCl. The samples were then centrifuged for 10 minutes at 10,000 rpm. The total collagen content in the supernatant was measured by the Sircol Collagen Assay Kit (Biocolor, Newtown Abbey, Northern Ireland, UK). The calcium content in the supernatant was measured by Calcium E-test WAKO (Wako, Tokyo, Japan). The results were expressed as mg collagen/mg dry weight and μg calcium/mg dry weight.

#### Histological analysis.

The mice for histological evaluation were killed by perfusion-fixation with 4% paraformaldehyde. The gastrocnemius muscles were embedded in Tissue-Tek OCT compound (Sakura Finechemical, Tokyo, Japan), and frozen in isopentane chilled with liquid nitrogen. Frozen non-decalcified sections were made with a cryostat (Bright, UK). The plane of the sections was axial (thickness 7 μm). BMP-4 expression was detected immunohistochemically. The primary antibody for BMP-4 (Anti BMP 2/4 (H-51), Santa Cruz Biotechnology, Santa Cruz, CA) was diluted at 1:100. As a negative control, 10% normal rabbit serum (Nichirei, Tokyo, Japan) was used. The slides were washed and exposed to horseradish peroxidase-conjugated corresponding secondary antibodies (DakoCytomation, Glostrup, Denmark) diluted at 1:200 for 1 hour at room temperature. Color development was then carried out with 3′,3′-diaminobenzidine tetrahydrochloride solution (Sigma Ltd, UK). Corresponding slides were stained with hematoxylin-eosin and with silver nitrate solution (von Kossa).

### Statistical analysis

The results of the radiographic analysis (bone area), total collagen, and calcium content were compared through a paired Student's *t* test. All data are expressed as the mean ± SD. *P* < 0.05 was considered to be significant.

## Results

### In vitro studies

#### Effect of LIPUS stimulation on the BMP-4 gene expression.

**Figure 1.**
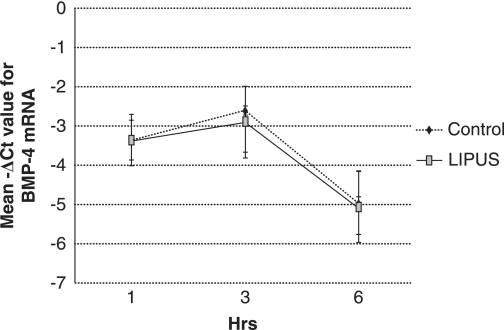
Effect of low-intensity pulsed ultrasound (LIPUS) stimulation on the *in vitro* bone morphogenetic protein (BMP)-4 gene expression. Each value represents the mean ± SD of three separate experiments. There was no significant difference in the expression of BMP-4 between experimental and control samples at all time points.

The expression of the mRNA for BMP-4 in C2C12 cells was determined by real-time PCR. There was no significant difference in the expression of BMP-4 between experimental and control samples at all time points (Figure 1).

### In vivo studies

All animals (*n* = 36) survived without any complications.

#### Expression of BMP-4.

 BMP-4 expression was immunohistologically observed at Day 7, 10, and 14, and not detectable at Day 21 (data not shown). Negative controls did not show any staining. The positive signals for BMP-4 were localized in the muscle fibers. The highest level of BMP-4 expression was seen at Day 7. The differences in BMP-4 expression between LIPUS and control sides could not be seen at any time point.

#### Histological examination.

**Figure 2.**
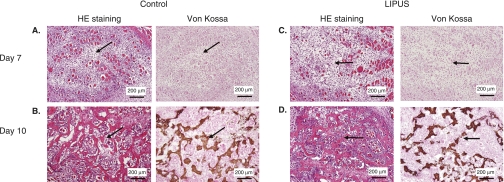
Histological examination of ectopic bone formation. A, B: control side; C, D: low-intensity pulsed ultrasound (LIPUS) side; A, C: Day 7; B, D: Day 10; (left) hematoxylin-eosin staining; (right) von Kossa staining. In both control side (A) and LIPUS side (C), mesenchymal like cells (arrows) were noted between muscle fibers but presence of ectopic bone formation was not seen in both sides at Day 7 (A, C). Ectopic bone formation (arrows) was clearly seen in both sides at Day 10 (B, D). Histological examination showed no obvious difference between LIPUS and control sides.

In both LIPUS and control sides, mesenchymal-like cells were noted between muscle fibers at Day 7 (Figure 2A, C, arrows). Ectopic bone formation was seen in both sides at Day 10 (Figure 2B, D, arrows), 14, and 21. Hematoxylin-eosin staining showed osteoid trabeculae of woven bone. The bone was surrounded by native myofibers. Von Kossa staining revealed bone mineralization within the gastrocnemius in both control and LIPUS side at Day 10 (Figure 2B, D), 14, and 21. Histological characteristics of the ectopic bone were similar between LIPUS sides and control sides at all time points.

#### Radiographical analysis.

**Figure 3.**
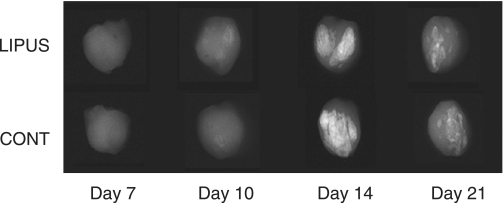
Soft X-ray images of the gastrocnemius muscles that were either low-intensity pulsed ultrasound (LIPUS) side (top) or control side (bottom) at different time points. At Day 7, there was no evidence of bone formation in either side. At Day 10, ossification areas became detectable in both sides. The ossification areas were the largest and most clearly visible in all animals in both sides at Day 14, and they became faint at Day 21.

**Figure 4.**
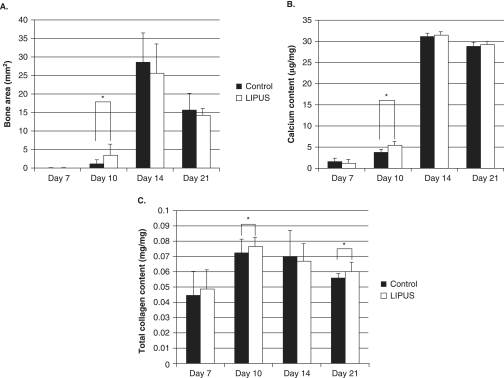
Areas of bone formation on radiogram (A), calcium content (B), and total collagen content (C) between control and low-intensity pulsed ultrasound (LIPUS) sides at each time point. Each value represents the mean ± SD. At Day 10, areas of bone formation on radiogram were significantly larger in the LIPUS side than that of in the control (*P* = 0.049). There was a significant increase in the calcium content with LIPUS exposure at Day 10 (*P* = 0.008). The total collagen content with LIPUS exposure was increased significantly over control at Day 10 (*P* = 0.022) and at Day 21 (*P* = 0.049). *P < 0.05 for LIPUS side versus control side.

At Day 7, there was no evidence of bone formation in either side. At Day 10, ossification areas became detectable in both sides. The ossification areas were the largest and most clearly visible in all animals in both sides at Day 14, and they became faint at Day 21 (Figure 3). At Day 10, areas of bone formation on radiogram in the LIPUS side were significantly larger than that in the control side (*P* = 0.049). The area of bone reached the maximum in both sides at Day 14. However, no significant difference between LIPUS and control sides could be detected at Day 14 (Figure 4A).

#### Quantification of calcium and total collagen content.

There was a significant increase in the calcium content with LIPUS exposure at Day 10 (*P* = 0.008). However, there were no significant differences in the calcium content at Day 7, 14, and 21 (Figure 4B). The total collagen content with LIPUS exposure was increased significantly over control at Day 10 (*P* = 0.022) and at Day 21 (*P* = 0.049). There were no significant differences at day 7 and 14 (Figure 4C).

## Discussion

The variant levels of BMP-4 expression from pCAGGS expression vector could affect the maturation and size of ectopic bone. The expression vector pCAGGS used in this study contains a cytomegalovirus immediate-early enhancer/modified beta actin promoter sequence. The gene-transferred cells ubiquitously express gene products under the regulation of this promoter. Our *in vitro* study confirmed that LIPUS stimulation has no influence on the expression of BMP-4 gene (Figure 1). In our immunohistological examinations, the differences in BMP-4 expression between LIPUS and control sides were not seen at any time point (data not shown). So, LIPUS is less likely to have an effect on BMP-4 expression *in vivo*.

In this study, we found LIPUS increased bone area and calcium deposition at Day 10 (Figure 4). According to *in vitro* studies on osteoblasts, biological effects of LIPUS have been evidenced. Warden et al. showed that LIPUS stimulated the expression of c-fos and cyclo-oxygenase-2 genes and elevated mRNA levels for the bone matrix proteins alkaline phosphatase and osteocalcin (26). LIPUS stimulates osteoblast differentiation (27) by increasing alkaline phosphatase, osteocalcin, and vascular endothelial growth factor expression and mineralization (28). These studies showed that ultrasound stimulated osteoblasts, activated bone matrix formation, and, finally, facilitated bone formation. In addition, we found that LIPUS caused a significant increase in total collagen content at Day 10 and 21 (Figure 4C). There are only *in vitro* studies of the influence of LIPUS on collagen synthesis during osteogenesis. Gileizal et al. reported that the expression of Collagen I gene was increased after stimulation by LIPUS in cultured osteoblasts (6). Sato et al. reported LIPUS up-regulated lysyl oxidase mRNA expression and elevated the total amount of cross-link formation of collagen on a MC3T3-E1 osteoblasts culture model (29). Our results are consistent with previously reported abilities of LIPUS to promote collagen synthesis and/or inhibit collagen degradation. This is the first report to demonstrate that LIPUS increases the collagen content in the *in vivo* bone formation model.

In the present study, LIPUS did not have a beneficial effect on the maximum size of ectopic bone at Day 14. In a rabbit distraction osteogenesis model, LIPUS led to an increase in bone mineral density and mechanical strength at 1 to 3 weeks, but they were similar in control and LIPUS groups by 4 weeks (30). Our results in ectopic bone formation are consistent with the results in the distraction osteogenesis model. The energy used for LIPUS treatment can only cause extremely low pressure waves at a rate proportional to the density of the tissue. LIPUS may be mainly reflected at tissue borders of soft and hard tissue, like connective tissue and cortical bone. This may be supported by the inability of LIPUS to stimulate osteogenesis in intact bone (31). At Day 14, cortical bone has already formed in the present study. LIPUS might not be able to cause further beneficial effect after cortical bone formation.

The size of ectopic bone reached the maximum at Day 14 and decreased at Day 21 in both LIPUS and control side (Figure 4A). It is reported that the amount of gene expression, induced by electroporatic gene transfer *in vivo*, decreases in 2–3 weeks (15,32). Our immunohistochemical evaluation also showed the depletion of BMP-4 production at Day 21. According to Wolff's law, without mechanical load the bone undergoes absorption (33). The ectopic bone induced in the muscle was not exposed to mechanical stress of loading. Therefore, the absorption of induced bone is a rather convincing result. Further study is necessary for the orthotopic bone formation induced by electroporatic transfer of BMP-coding genes.

Immunohistochemical analysis of the gastrocnemius muscle demonstrated the successful BMP-4 gene transfer into the skeletal muscle. BMP-4 protein seems to be localized only in muscle fibers. These results suggest that BMP-4-positive muscle fibers play an important role as preserver for a continuous supply of BMP-4 to the surrounding tissues. In addition, we have modified several parameters of the BMP-4 gene transfer model in this study. These modifications allowed the increase of successful bone induction rate from 67% in our previous study to 100%. C57BL/6J mice are reported to be a better responder for BMPs than the BALB/cA strain (34). Using this C57BL/6J strain and the pCAGGS expression vector, featuring higher activity in muscle (35), ectopic bone formation was observed in all animals at 14 days after electroporatic gene transfer (15). Also dystrophic calcification was seen after electric pulses in BALB/cA mice (14). A radio-opaque area was not observed in the gastrocnemius of C57BL/6J electroporated with pCAGGS-GFP, a GFP-containing plasmid, used as the control (15). Under refined experimental conditions, the dystrophic calcification by electroporation was assumed as negligible.

This study has technical limitations. The effect of LIPUS on ectopic bone formation was verified only by X-ray analysis and quantification of calcium and collagen content. The gene expression analysis could be more indicative of LIPUS-enhanced ectopic bone formation and subject to our future studies involving evaluation of ectopic bone formation.

In summary, our results show that LIPUS did not increase maximum bone volume in ectopic bone formation model *in vivo*, but in early phase LIPUS increased calcium and total collagen content and bone area. This is the first report to demonstrate that LIPUS increases the collagen content in the *in vivo* ectopic bone formation model.
